# Estimating causes of death where there is no medical certification: evolution and state of the art of verbal autopsy

**DOI:** 10.1080/16549716.2021.1982486

**Published:** 2022-04-04

**Authors:** Daniel Chandramohan, Edward Fottrell, Jordana Leitao, Erin Nichols, Samuel J. Clark, Carine Alsokhn, Daniel Cobos Munoz, Carla AbouZahr, Aurelio Di Pasquale, Robert Mswia, Eungang Choi, Frank Baiden, Jason Thomas, Isaac Lyatuu, Zehang Li, Patrick Larbi-Debrah, Yue Chu, Samuel Cheburet, Osman Sankoh, Azza Mohamed Badr, Doris Ma Fat, Philip Setel, Robert Jakob, Don de Savigny

**Affiliations:** a Department of Infectious and Tropical Diseases, London School of Hygiene and Tropical Medicine, London, UK; b Institute for Global Health, University College London, London, UK; c World Health Organization Verbal Autopsy Reference Group Secretariat, Luanda, Angola; d Centers for Disease Control, National Center for Health Statistics, US Public Health Service, Hyattsville, MD, USA; e Institute for Population Research and the Department of Sociology, Ohio State University, Columbus, Ohio, USA; f Department of Data Analytics and Delivery for Impact, World Health Organization, Geneva, Switzerland; g Department of Epidemiology and Public Health, Swiss Tropical and Public Health Institute, University of Basel, Basel, Switzerland; h Consultant, Saint-Legier, Switzerland; i Vital Strategies, New York, USA; j Department of Environmental Health and Ecological Services, Ifakara Health Institute, Dar Es Salaam, Tanzania; k Department of Statistics, University of California, Santa Cruz, USA; l Ghana Ministry of Health, Ghana Health Service, Accra, Ghana; m Ministry of Health, Nairobi, Kenya; n Statistics Sierra Leone, Freetown, Sierra Leone; o Heidelberg Institute of Global Health, Heidelberg Institute of Global Health, Heidelberg, Germany; p School of Public Health, University of the Witwatersrand, Johannesburg, South Africa

**Keywords:** Mortality surveillance, Civil Registration and Vital Statistics Systems, InterVA, InSilicoVA, SmartVA

## Abstract

Over the past 70 years, significant advances have been made in determining the causes of death in populations not served by official medical certification of cause at the time of death using a technique known as Verbal Autopsy (VA). VA involves an interview of the family or caregivers of the deceased after a suitable bereavement interval about the circumstances, signs and symptoms of the deceased in the period leading to death. The VA interview data are then interpreted by physicians or, more recently, computer algorithms, to assign a probable cause of death. VA was originally developed and applied in field research settings. This paper traces the evolution of VA methods with special emphasis on the World Health Organization’s (WHO)’s efforts to standardize VA instruments and methods for expanded use in routine health information and vital statistics systems in low- and middle-income countries (LMICs). These advances in VA methods are culminating this year with the release of the 2022 WHO Standard Verbal Autopsy (VA) Toolkit. This paper highlights the many contributions the late Professor Peter Byass made to the current VA standards and methods, most notably, the development of InterVA, the most commonly used automated computer algorithm for interpreting data collected in the WHO standard instruments, and the capacity building in low- and middle-income countries (LMICs) that he promoted. This paper also provides an overview of the methods used to improve the current WHO VA standards, a catalogue of the changes and improvements in the instruments, and a mapping of current applications of the WHO VA standard approach in LMICs. It also provides access to tools and guidance needed for VA implementation in Civil Registration and Vital Statistics Systems at scale.

## Introduction

Over the past 70 years, significant advances have been made in determining the causes of death in populations that are not served by official medical certification of cause at the time of death. This is achieved by the widespread application of a technique known as Verbal Autopsy (VA). VA is a methodology whereby a questionnaire is administered to the family or caregivers of the deceased after a suitable bereavement interval. The questionnaire probes the circumstances, signs and symptoms of the deceased in the period leading to death. The information obtained from the VA interview is then independently interpreted by a panel of physicians or, more recently, by a computer algorithm, to assign a probable underlying cause of death.

Most of these methodological advances in VA have occurred in the last 20 years in a period benefiting from innovations and efforts of the late Prof. Peter Byass and his colleagues. The current COVID-19 pandemic has brought into vivid focus the overall weaknesses of mortality surveillance in low- and middle-income countries (LMICs) and globally; furthermore, it has led to a growing demand for solutions like VA that can be applied widely in Health Information Systems (HIS) and in Civil Registration and Vital Statistics (CRVS) systems to address the deaths that occur in the most disadvantaged populations. These advances in VA methods are culminating this year with the release of a new World Health Organization (WHO) Standard Verbal Autopsy (VA) Toolkit. Highlighting the contributions of Peter Byass to this field, this paper briefly traces the evolution of VA and focuses on the latest development of the 2022 WHO Standard Instrument, the developments in methods for assigning the cause of death (COD) using VA data collected by the instrument, and implications for LMICs.

## Evolution of Verbal Autopsy instruments and standards

**Figure 1.**
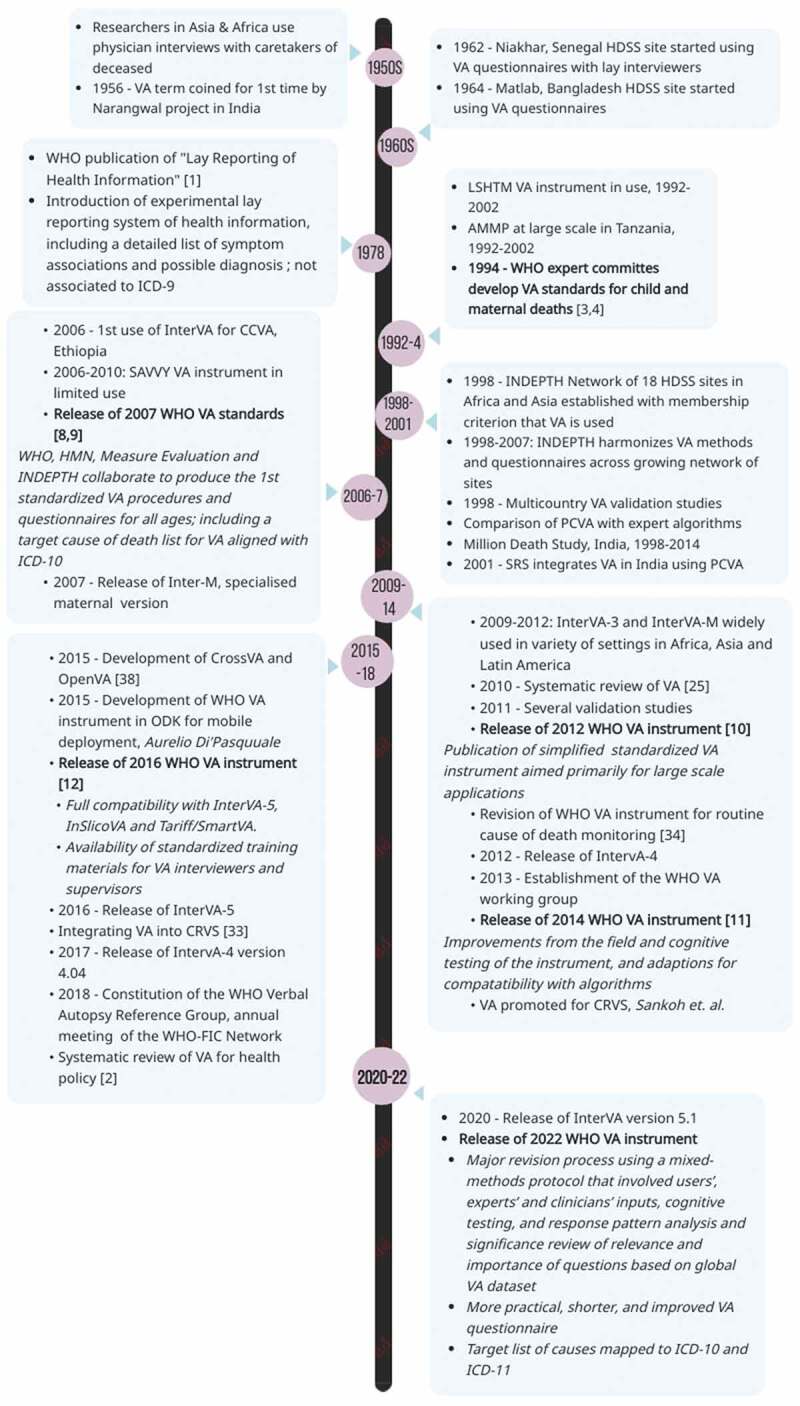
Timeline of milestones in the evolution of verbal autopsy standards.

The development of VA can be traced back to work in Asia and Africa in the 1950s and 1960s, where physician interviews with caretakers of deceased persons were conducted to assess the causes of death (Figure 1). In 1956 at the Narangwal project in India, the method was for the first time named ‘verbal autopsy’. Thereafter, it disseminated and developed, especially in the 1970s, when WHO promoted the use of lay reporting of health information by people with no medical training [1]. Several VA instruments were developed in research settings and used in national/regional surveys, and much effort was allocated into developing and refining the approach for specific objectives [2]. In the early 1990s, concerns arose about the validity of the many disparate instruments used and the comparability of generated data. This led WHO to convene expert committees to develop VA standards for childhood and maternal deaths in 1994 [3,4].

In 2006, a review of VA methods showed that VA was being routinely used as a research tool at small geographic scales in over 35 Demographic Surveillance Sites (DSS), mostly in Africa and Asia, in the Sample Registration System (SRS) sites in India and the Disease Surveillance Points (DSP) system in China [5]. The review also revealed that up to 18 distinct VA instruments were in use in 13 countries. The structure and content of the VA instruments, the target cause of death lists, field operating procedures and cause of death assignment processes were found to vary significantly among sites, highlighting the need to have reliable, harmonized, and standardized VA procedures to enable accurate national and international analysis and use of VA data [6].

In response to the proliferation and diversity of instruments and the need for standardization, the WHO asked an expert group of researchers, data users, and other stakeholders to systematically review the accumulated experience and evidence from the most widely used and validated methods, for developing standardized VA procedures. Of particular influence on the 2007 WHO VA standards was the VA instrument developed by the London School of Hygiene and Tropical Medicine (LSHTM) and that developed by the Adult Morbidity and Mortality Project (AMMP) in Tanzania [7]. In 2007, WHO published the first international VA standards for the ascertainment of causes of all deaths to further harmonise data collection, comparison and analysis [8]. These standards included:

- Verbal autopsy instruments for three age groups (under 4 weeks; 4 weeks to 14 years; and 15 years and above);
- Cause-of-death certification and coding resources consistent with the International Classification of Diseases and Related Health Problems, Tenth Revision (ICD-10); and
- A target cause-of-death list for VA aligned with the ICD-10.

The WHO standards outlined a cause of death assignment process that relied on three physicians trained to assign causes of death from VA. Physicians would independently interpret individual VA interview data and assign a cause of death. If there was agreement between at least two physicians, the cause of death was assigned accordingly. This is known as physician-certified VA (PCVA) [9]. This procedure had been in use by the International Network for the Demographic Evaluation of Populations and Their Health in Developing Countries (INDEPTH) and by the Sample Vital Registration with Verbal Autopsy (SAVVY) [3].

Following the first iteration of the standardised VA instrument and procedures in 2007, and in recognition of the need for large-scale use of VA for overcoming the scarcity of mortality data from many LMICs, subsequent updates of the VA standards were published in 2012, 2014 and 2016 [10–12]. The updates aimed at generating more simplified and practical instruments for routine and large-scale use, including national CRVS systems. The simplification process included modifying questions from the 2007 version to facilitate dichotomous yes/no responses (or some multiple pre-specified responses) and reducing the number of questions by restricting them to indicators that were deemed to be essential for assigning a cause of death by computerised algorithms or PCVA. The updates also promoted the development of automated methods for data collection and for assigning causes of death, to improve data consistency, comparability, validity and timeliness.

The first automated method to assign causes of death from VA available in the public domain was the InterVA suite of models developed by Peter Byass. The development of InterVA started in 2003 and the first version, InterVA-1, was released in 2005. The publication of the 2012 WHO VA Instrument concurred with the release of the InterVA-4 model that incorporated the 245 indicators included in the WHO VA instrument to assign one of the 62 ICD-based target cause categories from the WHO VA causes of death list [13]. This process signalled a significant transition from the previous uses of VA, which were generally restricted to small-scale research and surveillance settings towards routine use and were paralleled by a growing global momentum to strengthen CRVS systems in low-income countries [14]. The growing momentum also raised some key issues like ethical considerations, such as informed consent of the respondent, confidentiality of information, and ownership of VA data. Peter Byass provided significant contributions to these first standards, drawing from his extensive experience supporting VA within Health and Demographic Surveillance (HDSS) sites since 2003.

The year 2014 marked the start of a new iterative process of improvements that balanced measurement performance of VA instrument and its compatibility with algorithms for computer-coded verbal autopsy (CCVA). These efforts involved incorporating feedback and recommendations from field experience and cognitive testing of the WHO 2012 instrument conducted in Kenya, and adding or modifying questions to facilitate the use of publicly available automated analytical software for assigning the cause of death [15].

Again in 2015, it was observed by Byass and colleagues that there was still significant methodological heterogeneity in VA data collection and interpretation which amplified uncertainties over cause-specific mortality fractions [13]. With the goal of a global standard for reporting VA results, the need for a single standard VA instrument on which multiple diagnostic methods could be applied was recognized [16]. In 2016, WHO and global partners revised the WHO VA Standards to allow full compatibility with the CCVA algorithms that were available in the public domain, InterVA-5, InSilicoVA and Tariff/SmartVA [17]. This period coincided with emerging concerns about the feasibility and sustainability of PCVA when VA is applied on a large scale.

## Development of the 2022 WHO VA Instrument and Standards

**Figure 2.**
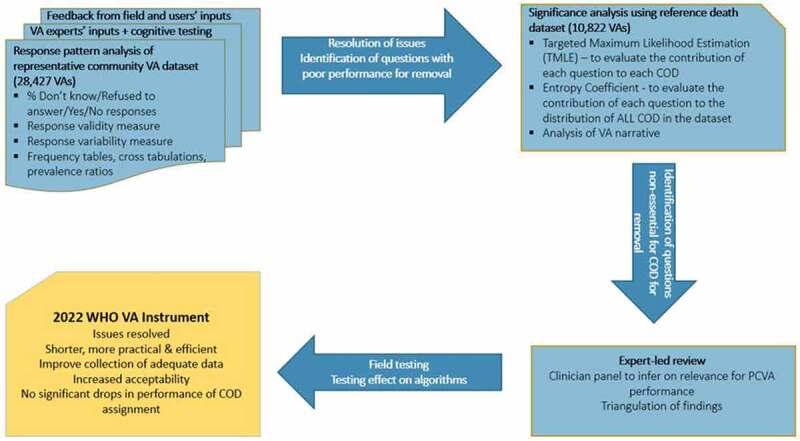
2022 WHO VA Instrument revision process.

Since its release, the 2016 WHO VA Instrument and Standards have been subjected to testing and extensive field use. An issue tracker was set up on the GitHub Platform^1^^1^GitHub is a web-based version-control and collaboration platform for software developers. (https://github.com/SwissTPH/WHO_VA_2016) where the users of the WHO VA instrument report problems they have faced and/or suggestion to improve. Users could report an issue through the web page or via email to whova@swisstph.ch. All issues reported to GitHub were compiled and were addressed as part of a major revision of the VA instrument carried out in 2020–2021 based on comprehensive users’ feedback and evidence from the field. The WHO VA Reference Group (WHO VARG), of which Peter Byass played a key role, used a mixed-method approach protocol (Figure 2), to produce an instrument that is as short, concise and efficient as possible, and that works well with currently available CCVA algorithms and PCVA.

In this mixed-methods approach, firstly, inputs from VA users and experts were used in combination with response pattern analyses of VA interviews from 28,427 community deaths in 13 countries^2^^2^De-identified data that contributed to the revision process of the 2021 WHO VA Instrument was provided from the following countries: Bangladesh, Ethiopia, Burkina Faso, Mali, Sierra Leone, South Africa, Ivory Coast, Ghana, Kenya, Mozambique, Morocco, Zambia and Thailand. to assess the usefulness of each indicator included in the VA instrument. Secondly, results of cognitive testing studies conducted in Morocco and Zambia were used to identify questions that could or could not obtain reliable responses from VA interviews. The outputs from these two steps were applied to resolve known issues from user experience with the 2016 WHO VA instrument. Examples of known issues include whether the open narrative should precede or follow the structured questions with selective responses; the degree to which the questionnaire continues once it is established that the death is due to external (injury) events; and issues concerning the time duration of signs and symptoms prior to death.

Thirdly, VA interview data from 10,822 VA’s along with a reference cause of death assigned by PCVA from Bangladesh, Ethiopia, Kenya, Mali, Mozambique, Sierra Leone, South Africa, Ghana and Thailand that were available in a WHO repository, were used to identify questions that are not providing useful information to assign the cause of death. Using the physician-assigned causes of death in the reference dataset, Targeted Maximum Likelihood Estimate^3^^3^Targeted Maximum Likelihood Estimation measures the contribution of each VA question to the determination of a single cause of death. (TMLE) [18] and Entropy Coefficient^4^^4^The Entropy Coefficient measures the contribution of each VA question to the determination of all causes of death. [19] were calculated to assess the importance of each symptom/sign in differentiating between causes of death. Neither of these procedures used any of the existing CCVA algorithms.

The fourth step of this review process involved an expert-led review (including a panel of clinicians experienced in PCVA) of all the findings to ensure that dropping any questions from the instrument would not reduce the performance of PCVA.

Finally, a series of virtual workshops involving VA experts and users was conducted to review and triangulate the results of the different analyses, reach consensus on solutions, and generate a list of questions recommended for revision or removal from the 2016 WHO VA instrument (Figure 2).

Outcomes from the revision process include a reduction in the number of questions and improvements in the structure and flow of the questionnaire as well as enhanced clarity and intent of questions. These adaptations are expected to lead to a shorter and more efficient interview process. However, there will be some reduction in the amount of information available to assign causes of death by CCVA and this may have an effect on the performance of the existing CCVA algorithms. In order to mitigate any reduction in the performance, minor adjustments may need to be done to the exiting CCVA algorithms. In the near future, when more data from the new instrument become available, CCVA algorithms can be modified further to improve the overall performance and efficiency of VA implemented at scale. The key features of the WHO 2022 VA instrument and standards are summarised in Tables 1 and 2. As of the time of publication, the 2022 WHO VA revised instrument is being programmed electronically with Open Data Kit (ODK) (https://getodk.org) ahead of field-testing scheduled for late 2021.

**Table 1.** Summary of features for the 2022 WHO VA Instrument

General Features	
Instruments, software and training materials	Available for download from WHO (footnote)
Deployment versions	Paper and Tablet (ODK)
Languages	English, Arabic, French, Kiswahili, Portugese, Spanish
General identification and context indicators	44
Age specific modules	Neonatal: 0–27 completed days (Under 4 weeks)
	Child: 28 completed days to 11 years (4 weeks to 11 y
	Adult: >11 years (12 years and above)
Number of indicators^a^	Neonatal 80; Child 133; Adult 179
Median time for interview^b^	Neonatal 19 mins; Child 27 mins; Adult 32 mins
Health service use during the fatal illness	Included
Health care treatment & experience before death	Included
Open narrative checklist	Included
Open narrative text	Included, located at the start of the interview
Open narrative audio	Included, located at the start of the interview
Status of Civil Registration of Death	Included
Status of Medical Certificate of Death	Included
Compliance with UN Statistics^c^	Yes
Batched analytics	Yes
Mapped to WHO ICD-10 & ICD-11 cause list	Yes
Mapped to IHME GBD cause list	Yes
Country applications of 2016 WHO VA in CRVS as of 2021	Bangladesh, Colombia, Ethiopia, Ghana, Kenya, Morocco, Mozambique, Rwanda, Senegal, Tanzania, Thailand, Zambia, and Zimbabwe

https://www.who.int/standards/classifications/other-classifications/verbal-autopsy-standards-ascertaining-and-attributing-causes-of-death-tool

^a.^Indicators managed by skip patterns. Categories overlap and are not mutually exclusive.

^b.^Based on the WHO 2016 instrument. Source: Mishra, V. (2017). Verbal Autopsy: Comparative analysis of three verbal autopsy algorithms with the WHO 2016 verbal autopsy questionnaire. MSc. Thesis, SwissTPH, University of Basel.

^c.^UN Fundamental Principles of Official Statistics compliance requires the use of software that provides open exchange of data and data processing techniques.

**Table 2.** Target causes of death for the 2022 WHO VA Instrument

Stillbirths	2 causes
Neonatal	7 causes
Maternal	12 causes
Communicable	17 causes
Non-communicable	22 causes
External (Injury)	11 causes
Total	71 with 64 discrete causes (overlapping categories)

## Approaches to assigning causes of death using WHO VA data

In research settings, verbal autopsy interview transcripts were often read by physicians who assigned the cause(s) of death (PCVA). To account for physician-specific bias, best practice PCVA requires that verbal autopsy questionnaire data are read by multiple physicians who together agree on a consensus cause(s). This approach to identifying a cause of death is not, in most cases, feasible or affordable for large-scale, routine mortality surveillance within health information or CRVS systems. In such large-scale applications, automated CCVA algorithms are used for assigning cause(s) of death. The most commonly used algorithms are InterVA/InSilicoVA [20,21] and SmartVA [22]. The key features of these algorithms are summarised in Table 3. These rely on a set of Symptom-Cause Information (SCI) [23] that describes the relationship between VA signs/symptoms and the causes of death on the list used by the algorithm.

**Table 3.** Comparison of specific features of diagnostic algorithms

Features	InterVA5	InSilicoVA	SmartVA/Tariff
Computing platform compatibility	Windows MacOs Linux	Windows MacOs Linux	Windows only
Number of indicators used by algorithm	304	304	211
Exact implementation/replication in openVA^a^	Yes	Yes	No
Implementation without training dataset	Yes	Yes	No
Can produce instantaneous results for a single death	Yes	No	Yes
Only significant symptoms used at individual level	No	No	Yes
Accounts for absence of symptoms	No	Yes	No
Accounts for missing symptoms	No	Yes	No
Provides distribution of probabilities for each cause for a single death	Yes	Yes	No
Provides measure of uncertainty for individual cause assignments	No	Yes	No
Direct estimation of cause-specific mortality fractions	No	Yes	No
Provides a distribution of probabilities for each CSMF	No	Yes	No
Provides uncertainty measure for cause-specific mortality fractions	No	Yes	No

^a^Source: Samuel J. Clark, openVA development team. www.openva.net.

Algorithms solve the problems related to physician bias, feasibility, and affordability. However, the accuracy of the causes of death assigned by them depends on the relevance and applicability of the SCI in a given context. For InterVA and InSilicoVA, the default SCI are conditional probabilities quantifying the frequency with which each symptom is expected to be present in a death due to the causes included in the target causes of death list. These probabilities are elicited from epidemiological evidence and physician consensus and stored in a matrix called ‘Probbase’. For SmartVA, the SCI serve the same purpose but are in the form of ‘tariff scores’ that are calculated directly from the Population Health Metrics Research Consortium (PHMRC) reference mortality dataset that has VA interview data and a reference cause of death list assigned by medical certification using clinical and laboratory results [24].

Strengths of the Probbase approach include: 1) it does not require expensive, logistically challenging reference deaths; and 2) that it draws on a wide, comparatively representative array of physician knowledge and experience. The main challenge is that it is inherently less precise because it is knowledge and experience filtered through individual human practice, rather than derived directly from data. The key strength of the Tariff approach is simultaneously its key weakness: the fact that it is drawn directly from the PHMRC reference death dataset ties it directly to a dataset of deaths in a limited number of hospitals in certain geographic and temporal settings (https://ghdx.healthdata.org). The tariff scores describe well the relationship between VA signs/symptoms and causes of death included in the SmartVA cause of death list but their applicability to other populations is debatable as they are derived from deaths that occurred in a limited time in six hospitals located in six geographic locations in three countries. Deaths occurring in the community outside hospitals at different locations and times are different in many ways from those included in the PHMRC reference death dataset. For example, few PHMRC deaths occurred in Africa and very few were affected by malaria, one of the most important causes of death in Africa.

The literature on comparisons of the performance of approaches to assigning causes of death from VA is hard to interpret. Proponents of the two main approaches, PCVA and CCVA, have attempted to elevate one or the other, and algorithm developers have done much the same for their algorithms. Evidence for the performance of each approach and the various algorithms is difficult to evaluate because studies do not use comparable data [23]. For assessing the performance of algorithms, only one study [21] applied all algorithms to the same deaths using the same SCI (all derived from the PHMRC reference death dataset); other studies have compared outcomes using different VA datasets and SCIs that render interpretation effectively impossible.

The reality is that assigning causes of death from VA is challenging, and there are strong advantages and disadvantages to all existing approaches. PCVA is potentially more accurate but also liable for physician-specific bias and low repeatability, and too slow and expensive for large-scale use. The probbase-driven algorithms benefit from SCI that is more general but less precise, while the tariff score-driven algorithms benefit and suffer from SCI that is closely tied to one, specific reference dataset. The empirically driven SCI is a more attractive solution, but only if it can be made more general and keeps up with the ever-changing epidemiology of populations where VA is required to determine causes of death.

A potential solution is to: 1) create a general, evolving, empirical SCI; and 2) enable fair comparisons between CCVA algorithms to develop a living reference death archive [25] hosted by a trustworthy third party, e.g. WHO. Such an archive would consist of a large and growing collection of reference deaths with standard VA signs/symptoms and a reference cause assigned through a reliable mechanism – importantly, not an algorithm – from a wide variety of settings through time. A key benefit of this WHO repository would be the potential to standardize the SCI used by all algorithms and thereby greatly increase the comparability of CCVA-assigned causes of death from VA. This would be invaluable for comparisons of all kinds, including tracking the burden of disease through time.

The choice of algorithmic approach best suited to any particular research or routine application is made easier by the fact that the WHO Standard Instrument is designed to work with all common algorithms. This allows implementers to compare results from all methods, consider their plausibility and utility along with the above considerations on their use of SCI, and the specific features outlined in Table 3 when selecting which method works best in their context.

## Peter Byass and the development of InterVA

In Peter Byass’s own words, ‘what we die from matters’ [20]. For this reason, Peter dedicated much of his scientific career to understanding and describing the causes of death among the world’s poorest populations who remain outside of formal systems of death registration and cause certification.

Peter recognised and articulated the need for fit-for-purpose tools to meet the differing needs of users of cause of death data [26]. Following decades of VA use in research contexts, debates around timeliness and reliability of PCVA, and limited success of alternative methods such as decision tree algorithms and neural networks, Peter conceptualised a new approach to assigning causes of death from VA [27]. He used a statistical approach proposed more than 300 years earlier by Thomas Bayes. Peter’s approach, known as InterVA (Interpreting VA) set in motion a renaissance of enquiry into VA methods and has been a leading force in the evolution of VA from a cumbersome surrogate for medical autopsy into a pragmatic, efficient tool for public health action.

InterVA was a huge breakthrough by quantifying the ‘likelihood’ of causes of death given reported signs and symptoms. These cause likelihoods are based on approximate probabilities of signs and symptoms being reported for each specific cause of death (*prior probabilities)*. Using Bayes theorem, it is possible to calculate the probability of each cause given the specific signs and symptoms reported. The ‘approximate’ nature of the prior probabilities might not sit well with some statisticians, but it simply is not possible to accurately know the prior probabilities for every sign and symptom for the causes of death of interest – certainly not in a population in need of VA. And herein lies the power behind Peter’s approach and his application of pragmatic solutions to real-world problems.

Initially, Peter estimated a set of prior probabilities based on accumulated personal experience, without any attempt to validate or establish internal consistency between estimated values. Using these estimated probabilities and a simple program created using FoxPro software, InterVA calculated probabilities for causes of death based on input signs and symptoms. This initial model was evaluated by comparing causes of 189 deaths from rural Vietnam assigned independently by InterVA and PCVA. In this comparison over 70% of individual causes of death corresponded with those derived from PCVA [28].

The next step was to apply epidemiological evidence and expert consensus to agree on, (1) a list of realistically identifiable causes from VA, (2) the signs and symptoms that could reasonably be expected to be recognised, remembered and reported by lay respondents in a VA interview and (3) estimated prior probabilities of each sign and symptom given a specific cause of death. The estimated prior probabilities were reviewed and revised by a panel of clinical and epidemiological experts representing different disease interests, medical specialities and global regions. The performance of the revised InterVA model was tested on 189 deaths from Vietnam. This showed the feasibility of InterVA for assigning causes of death and highlighted the advantages of speed, consistency and the ability to identify multiple possible causes for each death [29]. Subsequent application of the InterVA model in different settings and the development of a specific tool for pregnancy-related deaths [30], later combined with the full InterVA model, prompted further revisions and updates of the model, each requiring similar collaborative, consensus-building processes of reviewing and estimating prior probabilities. This refining process of InterVA not only created an improved tool but also stimulated new conceptualisations of ‘causes’ of death [31,32] debates around validity and ‘alloyed gold standards’ of reference causes of death [33]. It further provoked the linking of VA data collection and causes of death assignment processes as illustrated by the evolution of WHO VA standards over the past 15 years [20].

InterVA has become one of the most widely used methods for assigning causes of death from VA globally, and it contributed significantly to our understanding of the cause-of-death patterns in the world’s poorest populations. Peter’s work inspired other CCVA methods and sparked a broad body of academic enquiry into VA, combining disciplines of medicine, epidemiology, statistics, sociology, ethics and computer science.

Peter’s vision went far beyond academic enquiry. He was committed to capacity building and strengthening health systems through information. Available as a free-to-use, open-access software since its inception, Peter ensured InterVA was available for those who needed it. He led numerous training programmes and workshops through WHO and through the INDEPTH network of Health and Demographic Surveillance (HDSS) systems to ensure local, in-country capacity to process and manage their mortality data. His efforts strengthened individual’s and health system’s capacities and that’s one of the legacies of Peter’s work.

Peter Byass was actively contributing to the ongoing development of VA in response to the COVID-19 pandemic until shortly before his death. He had significant input into a ten-question stand-alone module and tool to assign causes of death to either ‘suspected COVID-19,’ ‘other natural causes,’ or ‘external causes.’ Subsequently, a revised set of these questions was incorporated into a special update of the 2016 WHO Standard VA Instrument.

## Application of Verbal Autopsy in LMICs

With the many methodological advancements in VA that Peter Byass supported, VA has become an increasingly important approach for investigating population cause of death data in contexts where deaths would otherwise be unknown without registration or certification. The use of VA in Health and Demographic Surveillance field sites in LMICs has been extensive, particularly for the production of aggregated COD statistics in populations without registration of individual deaths **[**34**]**. Attention is now focused on the incorporation of VA into Civil Registration and Vital Statistics Systems, as an interim approach to provide essential causes of death data, especially in rural populations, until medical certificates of causes of death are widely available. The use of effective data management practices can facilitate integration into ongoing death registration and reporting systems. Routine collection of causes of death through VA on all or a sample of community deaths analysed alongside causes of death data from hospitals can lead to more representative and usable population-level mortality statistics **[**35**]**.

### Use of WHO Verbal Autopsy standards in research, surveys and mortality surveillance in LMICs

**Figure 3.**
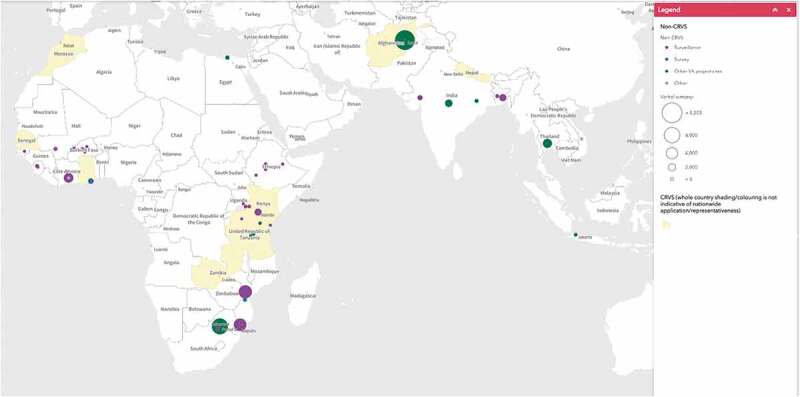
Map of applications of WHO Verbal Autopsy in research surveys, demographic surveillance (circles) and in national CRVS systems (country shading).

The INDEPTH Network, established in 1998, networked almost all HDSS sites in LMICs that were conducting VA in longitudinal studies of populations of at least 25,000 people. The purpose of INDEPTH was to better harmonize methods across the sites and to enable multi-site research projects using common protocols. As such, INDEPTH sites, of which there are currently 48 in 19 countries in Africa, Asia and Oceania (www.indepth-network.org), became early adopters of the WHO VA instrument and provided a testing bed for diagnostic algorithms, particularly InterVA and more recently InSilicoVA (Figure 3). InterVA, initially developed in INDEPTH sites in Ethiopia, South Africa and Vietnam by Peter Byass has become the most widely used approach to assigning causes of death from VA data in HDSS sites as they migrated their legacy systems from PCVA to CCVA analytics^5^^5^The placement of the circles in the map correlates with the geolocation of implementation sites and represents different implementation teams; whereas the size of the circles is proportional to the number of collected VAs using the 2016 WHO VA as reported by the teams. Shaded countries are in process of adding WHO VA to their CRVS system..

Beyond the large network of INDEPTH HDSS research sites, almost all new multi-country research projects and field trials that need cause-specific mortality data on large study populations are now using the 2016 WHO VA Standard or an adaptation of it along with its associated diagnostic algorithms. For example:

- MVIP, the WHO Malaria Vaccine Implementation Programme in Ghana, Kenya, and Malawi (https://clinicaltrials.gov/ct2/show/NCT03806465);
- CHAMPS, the Child Health and Mortality Prevention Surveillance in Bangladesh, Ethiopia, Kenya, Mali, Mozambique, Sierra Leone, and South Africa (https://champshealth.org)
- COMSA, the Countrywide Mortality Surveillance for Action in Mozambique and Sierra Leone (https://www.jhsph.edu/research/centers-and-institutes/institute-for-international-programs/current-projects/countrywide-mortality-surveillance-for-action-comsa-in-mozambique/).

At the single-country level, the 2016 WHO VA has been employed in national-scale research surveys such as a National Maternal Mortality Survey in Ghana, the Post Census Mortality Evaluation in Mozambique, and in a Demographic and Health Survey in Uganda.

### Use of WHO Verbal Autopsy standards in national Civil Registration and Vital Statistics Systems in LMICs

In LMICs, the WHO Standardized VA coupled with CCVA VA opens the door to take VA beyond the research domains mentioned above, to routine information systems at large scale [36]. Global and country-level efforts to improve CRVS systems are ongoing to ensure that every death is both counted and medically certified to its underlying cause. However, community deaths without proper medical certification will continue to occur for some time in many countries in LMICs. The demand for improved standardized VA tools and methods to improve mortality data systems will therefore continue. VA has significant potential in health policy and systems to deliver robust and reliable evidence, help close gaps in cause of death statistics, and guide implementation, evaluation and long-term investment strategies [37]. The strategy for VA within CRVS is to first register all community deaths by age and sex (cause marked as unavailable) and then generate representative cause of death distributions for the community deaths. Linking CRVS and VA data collection systems ensures that deaths are registered while also producing an ongoing stream of data on all-cause mortality and cause-specific mortality distributions. This system-wide approach requires a supportive legal and administrative framework, clear and efficient business processes, defined roles and responsibilities of all stakeholders, and an operational approach that is fully integrated into the operational architecture (including IT) of the HIS and CRVS [38]. The availability of standardized global and national resources including VA tools and accompanying manuals, and guidance documents on VA implementation has enabled countries to adopt and adapt them for use in their CRVS-VA scaling-up strategies [12,39]. Such resources include

- Integration of community-based VA into CRVS: System-level considerations;
- Training manuals for VA master trainers, supervisors and interviewers
- VA field interviewer manuals that include question by question guidance;
- ODK for VA: A quick guide;
- VA Sampling Strategy and Sample Size Calculator;
- VA Process Mapping Guidance;
- VA Costing Tool;
- Guidelines for Interpreting Verbal Autopsy Data;
- Guidance on the analysis of VA and MCCD data (under review).

Several countries supported by the Bloomberg Philanthropies Data for Health Initiative’s CRVS Improvement Program and other initiatives supported by development partners such as the Global Fund, World Bank, etc., are in the early stages of supporting scale-up of VA applications integrated into country HIS and CRVS systems. Countries in this early stage of CRVS-VA planning and integration using or considering WHO standard methods include: Bangladesh, Colombia, Ethiopia, Ghana, Kenya, Morocco, Mozambique, Rwanda, Senegal, Tanzania, Thailand, Zambia, and Zimbabwe (Figure 3).

As noted above, in several LMIC countries there is a history of existing HDSS or SAVVY sites using VA in research settings at sub-national scale. Where these sites use the WHO standard methods, they can provide useful expertise for planning and master training for VA in national CRVS scale-up.

Countries can adapt the WHO VA Standards for country-specific contexts to foster coordination and communication in support of system-wide VA application to improve population-level estimates of causes of death. This helps to avoid the proliferation of uncoordinated VA activities in the country that may derail national efforts to harmonize VA tools and methods for routine application (e.g. in Kenya [40]).

## Future developments of VA on the near horizon

A large community of partners with expertise in VA is now dedicated to supporting the continued improvement of VA instrument and procedures based on WHO standards. This includes maintaining a WHO web platform to serve the open availability of the 2022 WHO VA Instrument, its revisions as required, its training and management guides, and translations to more languages beyond the current English, Arabic, French, Swahili, Portuguese, and Spanish. The platform also serves as a two-way portal to receive inputs from field applications. It provides access to a GitHub portal to access the ODK versions of the Instrument (XLSForm https://xlsform.org/en/ a form standard created to help simplify the authoring of forms in Excel) for mobile deployment on Android devices in the field. The downloadable ODK XLSForm instrument after conversion to xml format (XForm), can be rendered across several web and mobile data collection platforms (e.g. Enketo, ODK Collect, etc.). The data collected via the web platform or offline android device can be sent in near-real-time to a central ODK server at national level to be analyzed.

To further support VA management in the field, partners have developed a tool to harvest VA interview data from ODK and prepare them for assigning causes of death using InterVA, InSilicoVA, or Tariff. (OpenVA) [41]. Managing large numbers of VA interviewers across a country in a national CRVS-VA enterprise is also supported by new tools such as VA Manager (https://crvs.moh.go.tz/download.jsp and VA Explorer (https://github.com/VA-Explorer/va_explorer) which link to DHIS2, a widely used digital HIS. Tools have also been developed to assist with VA data interpretation such as the VIPER tool (Verbal Autopsy Interpretation, Performance and Evaluation Resource (https://crvsgateway.info/VIPER).

Obtaining national or subnational cause-specific mortality fractions (CSMFs) does not require a VA on every death in a CRVS system. WHO recommends stratified, multi-stage, cluster, proportional to population size sampling. Partners have developed a VA Sample Size Calculator Tool and a Guidance document for selecting community clusters for nationally representative CSMFs with sufficient uncertainty ranges [12].

The COVID-19 pandemic in 2020 added another level of complexity to the field application of VA both in surveys and surveillance, but also in CRVS applications. Therefore, some countries are exploring alternative or complementary ways of conducting household level VA interviews in the event of a health crisis or other conflict and humanitarian crises in hard-to-reach places. One such approach is conducting VA interviews by telephone (TeleVA). While there have been few small-scale explorations and applications of TeleVA [42], further large-scale feasibility and acceptability studies on conducting VA interviews using mobile devices will need to be undertaken.

Recently an automated analysis of the open narrative to assign causes of death from VA has been developed. Until recently, the narrative report of the context and symptoms preceding the death recorded in the VA instrument was used in a complementary way by physicians to assign a causes of death in PCVA. The current revolution on deep learning neural models has opened the way to efficient and accurate text processing capabilities. To extract meaningful information from these unstructured texts, modern deep learning networks for natural language processing have been used with promising results [43]. Automatic examination of large volumes of data can be the key to infer models that adaptively show the evolution of causes of mortality in each region and age groups. When available, additional information such as medical record review summaries and pathology results from minimally invasive tissue samples (MITS) [44] could be included in the deep learning neural models.

WHO is supported by the Network for the Family of International Classifications (WHO-FIC), which includes collaborating centers, NGOs, nominated technical experts of member states, and other partners. The mission of the WHO-FIC Network is to improve health through the ongoing development, maintenance, and promotion of integrated suites of reference health classifications, terminologies and related products that produce information of value and utility across the world. The WHO VARG,^6^^6^VARG Members: Arvind Pandey, Aurelio Di Pasquale, Azza Badr, Carine Alsokhn, Carla AbouZahr, Chalapti Rao, Daniel Chandramohan (past Chair), Daniel Cobos (Co-chair), Debbie Bradshaw, Don de Savigny, Doris MaFat, Edward Fottrell, Erin Nichols (Co-Chair), Henry Kalter, Jordana Leitao, Lalit Dandona, Martin Bratschi, Pamela Groenewald, Philip Setel, Riley Hazard, Robert Jakob, Robert Mswia, Samuel Cheburet, Samuel Clark, Shams El Arifeen, Soewarta Kosen, Tita Rosita Wiguno, and Vishnu Vardhan. as part of the WHO-FIC Network, supports and advises WHO on the development and maintenance of the WHO Verbal Autopsy Instrument and supporting materials that complement causes of death determination in populations who are not served by official medical certification of cause at the time of death.

This paper has summarized the extensive developments of VA over the last several decades, highlighting the robust progress and standardization in the last 20 years that have been largely sparked by the vision and commitment that Peter Byass had to improve cause of death information among the world’s poorest populations. Owing much to Peter’s contributions, the field is well set to continue this trajectory in growth, reaffirming that ‘what we die from matters’.^7^^7^Provisional map elaborated with information provided by: the governments of India, Afghanistan, Nepal, Morocco, Ghana, Kenya, Mozambique, Rwanda, Senegal, Tanzania and Zambia; the HDSS sites of Nouna and Nanoro in Burkina Faso; the HDSS sites of Baliakandi and Matlab in Bangladesh; the HDSS site of Taabo in Ivory Coast; CHAMPS; COMSA; the HDSS site of Butajira in Ethiopia; Ghana’s DHS survey; the Malaria Research Centre; the HDSS site of Vadu in India; The University of Indonesia; KEMRI; the HDSS site of Kwale in Kenya; the HDSS sites of Manhiça and Chonkew in Mozambique; Kilamanjaro Christian Medical University College; Duke University; Africa Academy for Public Health; Ifakara Health Institute; the HDSS site of Kisesa in Tanzania; the HDSS site of Iganga-Myuge in Uganda; Alexandria University; the Aga Khan University; and South African Medical Research Council.

## Paper context

Recent advances in mobile information and communication technologies for surveys coupled with WHO standards are enabling the use of complex verbal autopsy data collection in remote settings with centralized automated analytics. This paves the way for widespread use of verbal autopsy in routine health information and CRVS systems in LMICs where medical certification of all deaths is not available. This improvement in supply-side for specific cause of death data coincides with an increasing demand for information to shape national policies and monitor progress on many Sustainable Development Goal Targets. This paper provides an overview of these advances and highlights the contribution of late Prof Peter Byass.
